# The Effectiveness of a Technology-Supported Behavioral Intervention Package for Teaching Earthquake Safety Skills to Children with Autism Spectrum Disorder: A Multiple-Probe Single-Case Evaluation

**DOI:** 10.3390/bs16101897

**Published:** 2026-10-14

**Authors:** Özge Ünlü

**Affiliations:** Department of Special Education, İzmir Demokrasi University, 35140 İzmir, Türkiye; ozgezybk@gmail.com

**Keywords:** autism spectrum disorder, behavioral intervention, single-case experimental design, safety skills, multiple-probe, Tau-U

## Abstract

Children with autism spectrum disorder (ASD) are at heightened risk during earthquakes, yet behavioral interventions targeting disaster preparedness in this population remain scarce. This study evaluated the effects of a multicomponent, technology-supported behavioral intervention package on the acquisition, maintenance, and generalization of earthquake safety skills—declarative knowledge of pre- and post-earthquake actions and a five-step drop–cover–hold chain under stimulus control—in children with ASD. Three male children with ASD (aged 8–12 years) took part in a probe-condition multiple-probe across-participants single-case experimental design. The package combined video modeling, slide-based concept presentation, conditional discrimination training, and in vivo rehearsal with least-to-most prompting and reinforcement schedule thinning. All three participants reached the maximum score after three to four instructional sessions and met the mastery criterion (three consecutive maximum scores) within six to seven daily probes. Performance was maintained at 2-, 4-, and 8-week follow-up probes, with one score of 4 at 4 weeks, and generalized to a novel setting, materials, and implementer. Visual analysis indicated three within-study replications of effect, one per participant, produced by the staggered introduction of the intervention across the three tiers of the design, and Tau-U effect sizes were 0.80 to 0.87 (large to very large). Within the limits of a three-participant design meeting What Works Clearinghouse standards with reservations, these findings indicate that structured behavioral instruction can establish earthquake safety responding in children with ASD; replication is required before wider instructional use.

## 1. Introduction

Earthquakes cause large-scale loss of life, property damage, and social disruption in seismically active regions (Centre for Research on the Epidemiology of Disasters [CRED], 2024). In Türkiye, the February 2023 Kahramanmaraş earthquakes killed more than 50,000 people and exposed serious gaps in disaster preparedness for vulnerable populations (Sarı, 2025). People with disabilities are at disproportionate risk in disasters: during the 2011 Great East Japan Earthquake and Tsunami, persons with disabilities were about twice as likely as others to lose their lives or be injured (United Nations International Strategy for Disaster Reduction [UNISDR], 2014). Children with autism spectrum disorder (ASD) can be at particular risk during such events. Differences in social communication, sensory processing, and the presence of restricted and repetitive behaviors (American Psychiatric Association, 2022) can make it harder to respond to safety instructions under high-stress conditions, particularly when disaster risk reduction does not address the barriers that people with disabilities face (United Nations, 2006; Villeneuve et al., 2021).

Children and adolescents with ASD may also be affected disproportionately by the consequences of an earthquake. Sensory processing differences in autistic children and adolescents are associated with heightened neurophysiological stress and anxiety (Pergantis et al., 2025). After the 2023 Kahramanmaraş earthquakes, scores for hyperactivity, lethargy, stereotypic behavior, and sleep anxiety increased among children and adolescents with ASD relative to pre-earthquake levels (Eroğlu & Yakşi, 2023), and parents of children with ASD and intellectual disabilities reported serious physical, psychological, and educational harm and an increase in behavior problems (Şahin & Kaya, 2025). After the 2009 L’Aquila earthquake, the adaptive behavior of children and adolescents with ASD declined markedly in the first months after the event (Valenti et al., 2012), and post-traumatic stress symptoms were more prevalent among children living near the epicenter (Feo et al., 2014). Disabled people affected by the Christchurch earthquakes linked their vulnerability to inadequate preparedness, inaccessible public information, and social isolation (Phibbs et al., 2014), and search and rescue personnel who worked with autistic individuals after the 2023 earthquakes in Türkiye described communication breakdowns, sensory and emotional distress, and uncertainty about how to interact with them (Tunç Paftalı et al., 2026). The needs of children and youth with disabilities are often not considered in emergency preparedness planning (Lindsay & Hsu, 2024; Stough & Kelman, 2018), which highlights the need for systematic instructional approaches tailored to learners with ASD.

The present study is grounded in applied behavior analysis, which supplies the conceptual framework for both the intervention and the measurement of its effects. Within this framework, an earthquake safety response is treated not as an item of knowledge but as a behavior chain: a sequence of discrete motor responses in which the completion of each step provides the discriminative stimulus for the next, with the chain as a whole evoked by a single antecedent event. Teaching such a repertoire therefore involves establishing antecedent stimulus control, so that the chain is occasioned by earthquake-related stimuli rather than by instructional cues; conditional discrimination training, so that the learner distinguishes safety-relevant from safety-irrelevant actions across varied exemplars; response prompting with systematic prompt fading, so that responding transfers from instructor-delivered prompts to the natural antecedent stimulus; contingent reinforcement with schedule thinning, so that responding is acquired under dense reinforcement and maintained under leaner conditions; and explicit programming for generalization, so that responding occurs across settings, materials, and persons not used during instruction rather than remaining bound to the training context (Cooper et al., 2020; Stokes & Baer, 1977). Each component of the package evaluated here was selected to serve one or more of these functions, and the outcomes examined—acquisition, maintenance, and generalization—correspond directly to them.

Empirical research on disaster preparedness instruction for children with ASD remains limited but is growing. Descriptive work has reported that disaster awareness training and simulation exercises increased preparedness among children with autism in a special education school (Indriasari et al., 2018), and a recent study combined applied behavior analysis with an immersive three-dimensional earthquake simulation (Ngoc et al., 2026). Three single-case experimental studies have evaluated instruction in earthquake safety responses. Kurt et al. (2024) evaluated video modeling for earthquake and post-earthquake evacuation safety skills with three children with ASD in a multiple-probe across-participants design and reported acquisition of basic protective behaviors (e.g., drop–cover–hold), generalization to a new setting, and maintenance at 4 weeks. Ulaşman and Sivrikaya (2025) used digital social stories to teach the drop, cover, and hold on technique and safe evacuation to three primary school students with ASD, with generalization to different settings and maintenance at 1, 3, and 5 weeks. Gürsoy and Kot (2026) used video modeling in a multiple-probe design across behaviors to teach two adolescents with ASD to prepare an emergency kit, perform drop, cover, and hold on, and move safely to an emergency assembly point, with maintenance and generalization to a different school setting. Notwithstanding these contributions, three gaps remain. They characterize the available evidence base rather than any single study: the experimental literature is small, and reviews of disaster preparedness for children and youth with disabilities describe a population whose needs are often left out of preparedness planning (Lindsay & Hsu, 2024; Stough & Kelman, 2018). First, the single-case studies described above measured overt safety responses; none reported a separate measure of declarative knowledge of pre- and post-earthquake actions within the same instructional protocol. Second, each evaluated a single primary instructional procedure, video modeling or digital social stories, rather than a multicomponent package that integrates technology-based modeling and concept presentation with conditional discrimination training and in vivo rehearsal. Third, maintenance has been reported over relatively short intervals (4 weeks in Kurt et al., 2024, and up to 5 weeks in Ulaşman & Sivrikaya, 2025), leaving maintenance over longer intervals largely unexamined.

These three gaps are consequential for both research and practice. Disaster preparedness for children with ASD requires not only motor execution of protective responses but also conceptual understanding of behaviors before, during, and after an earthquake. Multicomponent instructional packages that integrate diverse technology-based components may support attention and engagement for learners who benefit from visual and structured presentation of information. A broader body of work has examined technology-mediated instruction for children and adolescents with ASD, including robotics-based educational tools (Alghamdi et al., 2023) and immersive virtual reality applications for safety-relevant skills such as street crossing, driving, and bus riding (Liu et al., 2024). Video-based instructional procedures, in particular, have demonstrated efficacy in teaching a range of skills to individuals with ASD across settings. For example, Kaya and Yucesoy-Ozkan (2022) used self-directed video prompting with and without voice-over narration to teach daily living skills to students with ASD, showing that the procedure was effective for teaching, maintaining, and generalizing the skills both with and without narration. Similarly, Stauch and Plavnick (2020) employed video modeling within a multiple-probe design across behaviors to teach vocational and social skills to adolescents with ASD, providing evidence that video-based instructional packages can support skill acquisition under single-case experimental conditions. These findings support the rationale for evaluating a multicomponent package that combines video modeling with concept-level instruction, conditional discrimination, and motor rehearsal for teaching earthquake safety responses.

Four terms used throughout this report require definition. Earthquake safety skills are defined here as the combination of declarative knowledge of the actions to be taken before and after an earthquake, assessed through verbal and picture-based responding, and the overt motor repertoire to be executed during an earthquake, operationalized as the five-step drop–cover–hold chain. The intervention is described as technology-supported rather than technology-delivered because the digital materials—an animated film and a slide-based concept presentation—functioned as modeling and stimulus-presentation components within a package that also included instructor-delivered conditional discrimination training, in vivo rehearsal, prompting, and reinforcement; the technology supplied the antecedent stimuli, whereas the behavioral procedures established the response chain. A behavioral intervention package denotes the combined, simultaneously delivered set of these procedures, evaluated as a single independent variable without component analysis. Effectiveness is used in the restricted sense appropriate to a single-case experimental evaluation: a demonstration, replicated within the study across participants, that the targeted responses changed when and only when the package was introduced, together with evidence that the acquired responding persisted over time and occurred under untrained conditions. It does not denote comparative efficacy relative to an alternative intervention, nor effectiveness under routine service-delivery conditions.

The purpose of the present study was to evaluate the effects of a multicomponent, technology-supported behavioral intervention package on the acquisition, maintenance, and generalization of earthquake safety skills in children with ASD. The study addressed three research questions: (a) Does the behavioral intervention package increase declarative knowledge of pre- and post-earthquake safety actions in children with ASD? (b) Does the package establish overt protective behaviors (drop–cover–hold) under instructional and generalization conditions? (c) Are acquired skills maintained at 2-, 4-, and 8-week follow-up probes across novel settings, materials, and implementers?

## 2. Materials and Methods

### 2.1. Ethics

This study was conducted in accordance with the Declaration of Helsinki (World Medical Association, 2013). Ethical approval was obtained from the İzmir Demokrasi University Social Sciences and Humanities Ethics Committee (Protocol No. 2023/57, Decision No. 2023/08-10, dated 2 June 2023). Written informed consent was obtained from the parents or legal guardians of all participants. All procedures were designed to minimize potential discomfort and to respect participants’ rights. Written informed consent documents are securely stored and may be made available to the journal’s editorial office upon reasonable request. No modifications to the research protocol were made after approval was obtained.

### 2.2. Participants

Three male children with ASD, aged 8 years 7 months to 12 years 7 months, participated. Inclusion criteria were (a) a clinical ASD diagnosis confirmed by an independent clinician using Diagnostic and Statistical Manual of Mental Disorders (5th ed.; DSM-5) criteria, (b) ability to follow simple instructions (e.g., “come,” “take”), (c) sustained attention to visual and auditory stimuli for at least 10 min, (d) ability to respond to questions about a short story, and (e) no prior earthquake safety skills training. Prerequisite skills were verified through family interviews and individual assessment sessions conducted by the author. Participant characteristics are summarized in Table 1.

Utku was 8 years old and enrolled in an inclusive elementary school setting, receiving 15 h per week of resource-room instruction in a Special Education Class. His expressive language was generally limited to three-word phrases, and his speech was characterized by hypophonia. His reading performance was below grade level, and he required individual support in mathematics. He could focus on instructional tasks but required external prompts when his attention wandered. He showed a restricted interest in toy cars and exhibited stereotypic behaviors in certain situations.

Kaan was in the fourth grade and attended full-time inclusive education. His reading performance was slightly below grade level. He could perform basic arithmetic operations but required support with word problems. His communication profile included delayed and immediate echolalia. He frequently asked repetitive questions, particularly about car brands. Although he participated in classroom activities, he frequently left his seat and moved around the classroom.

Atakan was in the seventh grade at the start of the study. His overall academic performance was comparable to his peers, except in mathematics and science, which were slightly below average. His speech was intelligible but quiet. He had difficulty initiating and maintaining peer relationships. He exhibited a marked interest in soccer cards, which, when physically present, made sustained attention to other tasks difficult.

### 2.3. Setting and Materials

Sessions were conducted in a quiet classroom on the sixth floor of a university building in Türkiye. The controlled setting was selected to minimize sensory distractions during initial skill acquisition, consistent with recommended practice for instructional research with children with ASD. Generalization data were collected in the university’s ground-floor foyer, which differed from the instructional classroom in physical layout, lighting, ambient noise, and the presence of unfamiliar persons.

Materials included: (a) a 5-min animated film on earthquake safety produced by the Disaster and Emergency Management Presidency (AFAD), (b) an 18-slide PowerPoint presentation depicting illustrated earthquake safety protocols, (c) 40 visual cards (15 × 15 cm for pre- and posttest probes; 10 × 10 cm for generalization probes; see Table 2), (d) a laptop computer, (e) an illustrated booklet on earthquake actions, (f) two high-definition cameras for session recording, and (g) reinforcers (e.g., toy cars, crayons) selected through a paired-stimulus preference assessment conducted prior to baseline.

### 2.4. Experimental Design

A probe-condition multiple-probe across-participants single-case experimental design (Horner & Baer, 1978; Ledford & Gast, 2018) was used to evaluate the effects of the behavioral intervention package on the primary dependent variable (overt during-earthquake safety responses). In this design, the introduction of the intervention is time-lagged (staggered) across three participants (tiers) so that intervention is introduced sequentially rather than simultaneously: only one participant enters the intervention phase at a time, while the remaining participants remain in the baseline condition and are assessed in each probe condition. This staggered introduction across tiers, combined with stable baseline responding at zero level for each participant prior to intervention, provides the basis for demonstrating experimental control: a change in behavior is expected to occur in each participant only when, and after, the intervention is introduced to that participant, with no concurrent change in non-intervened participants. Three within-study replications of the predicted intervention effect, one per participant, were planned to satisfy the standards for documenting a functional relation in single-case research (Kratochwill et al., 2010; What Works Clearinghouse, 2020). Visual analysis of the data across five dimensions (level, trend, immediacy of effect, variability, and overlap) was supplemented with Tau-U effect size calculations to quantify the magnitude of intervention effects. Graphical presentation of single-case data followed established conventions for single-case line graphs in special education research (Kubina et al., 2021).

### 2.5. Dependent Variables

**Figure 1:**
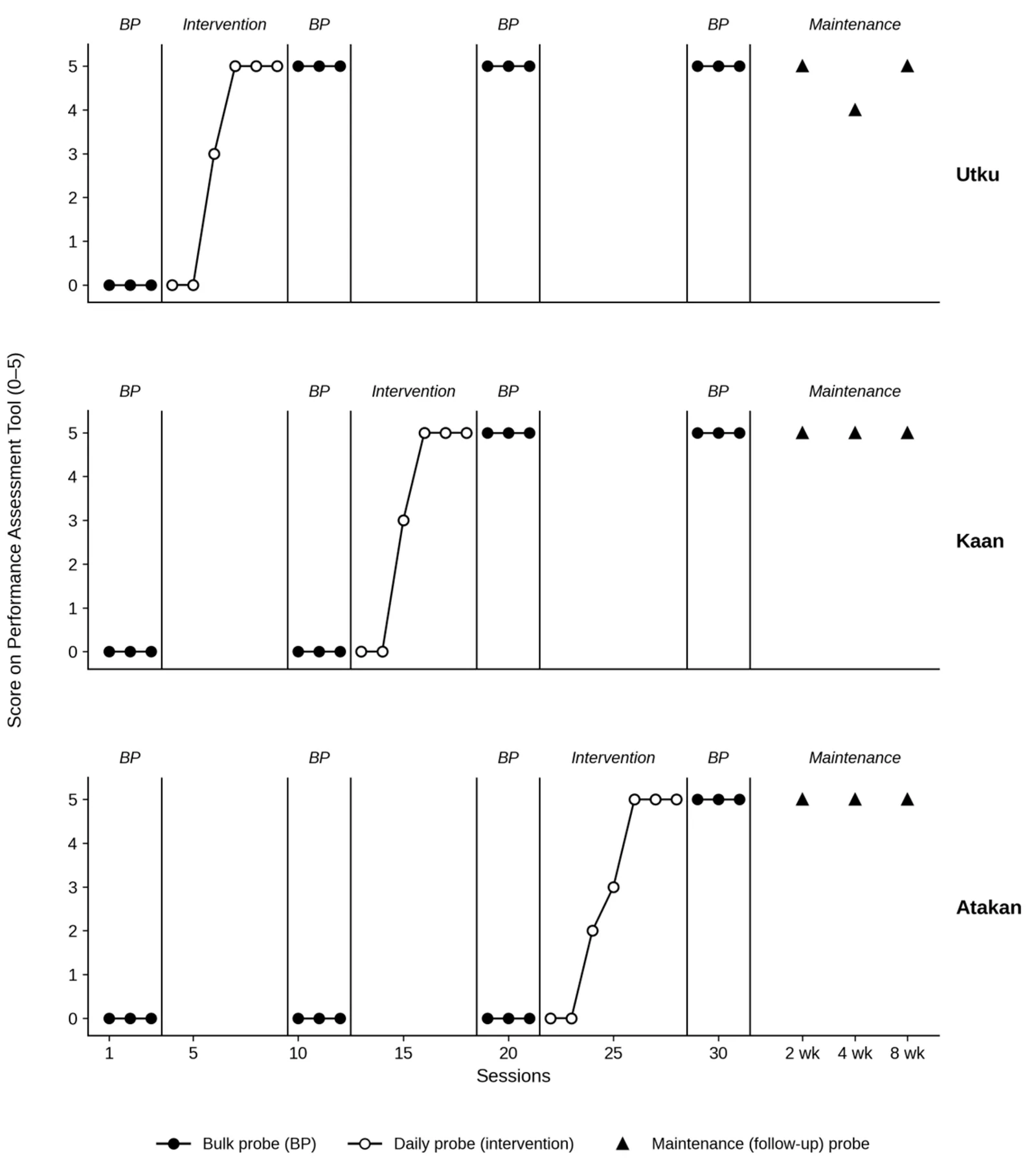
Probe-condition multiple-probe across-participants design. Performance scores (0–5) on the earthquake safety skill measure for each participant. BP = bulk probe condition (Probes 1–4; Section 2.9). Intervention = daily probes conducted at the start of each instructional session, before instruction (Section 2.10); the first intervention-phase data point for each participant was obtained before the first instructional session. Maintenance = follow-up probes at 2, 4, and 8 weeks after each participant’s own last instructional session, plotted after Probe 4 for clarity rather than at their chronological position. Vertical solid lines separate conditions. Closed circles represent probe sessions, open circles represent daily probes during the intervention phase, and closed triangles represent follow-up probes. Generalization sessions are reported separately (Section 3.4). Tau-U effect size values for each participant are reported in the Section 3.

The primary dependent variable was overt performance of during-earthquake safety behaviors, measured using a hierarchical 0–5 scoring system on the Performance Assessment Tool. The scoring system, summarized in Table 3, was cumulative: a score of 5 indicated the performance of all five target behaviors (staying calm, identifying the safe area, collapsing, closing into the fetal position, and holding on), and 0 indicated that Step 1 was not met, either because a behavior incompatible with safety occurred (e.g., panic, running toward exits) or because the participant did not initiate movement toward the safe area. Because the scoring system is hierarchical, intermediate scores reflect the performance of progressively fewer target behaviors. Performance scores were retained as raw scores on the 0–5 scale to preserve the hierarchical meaning of the measure and to avoid spurious precision introduced by percentage conversion. This variable was measured under the multiple-probe across-participants design with repeated probe-condition assessments at baseline, during the intervention phase (daily probes), post-intervention, and at follow-up (see Figure 1).

Because the first step in the response chain combined the absence of panic responses with the initiation of protective action, an explicit operational definition was established for scoring purposes. Staying calm was scored as present when, within the 5-s interval following the discriminative stimulus (“Earthquake!”), the participant initiated movement toward the designated safe area without emitting any of the following competing responses: sudden running, screaming, moving toward a window, door, staircase, or elevator, or climbing onto or jumping from furniture. Both conditions had to be met. Remaining motionless or waiting without initiating movement toward the safe area did not meet the definition, because the target was calm initiation of the protective sequence rather than the mere absence of agitation. Reaching and remaining within the safe area was scored separately as Step 2. The scale and this definition were reviewed with the second observer during observer training, and both observers applied the definition when scoring the video-recorded sessions for interobserver agreement (Section 2.13). Performance was scored with a single-opportunity method: the steps of the chain were scored in order, the assessment ended at the first step that was not performed correctly, and all remaining steps were scored as incorrect (Cooper et al., 2020). This method was chosen because multiple-opportunity probes, in which every step is assessed regardless of earlier errors, can themselves produce learning across repeated assessments of a chained task (Alexander et al., 2017). Under this rule, the cumulative score equals the number of consecutive steps performed correctly from the start of the chain; a later step could not be credited if an earlier step was omitted, and a separate step-by-step record would therefore contain no information beyond the cumulative score. Single-opportunity probes are also conservative, in that they may underestimate partial performance of the chain (Alexander et al., 2017).

Declarative knowledge regarding pre- and post-earthquake actions was measured as a secondary, supplementary descriptive variable to characterize changes in knowledge associated with the intervention. Because declarative knowledge was assessed as a discrete pretest-posttest-follow-up measure rather than through repeated probes consistent with the multiple-probe design, this variable is reported descriptively (see Section 3.1 and Section 3.2) and is not interpreted as providing experimental control. Knowledge-level performance was defined as the percentage of correct responses to questions presented with corresponding visual cards on the Knowledge Level Assessment Tool, with the 0–10 raw count converted to a percentage to facilitate comparison across the pre-earthquake and post-earthquake item subsets.

The Knowledge Level Assessment Tool and Performance Assessment Tool were developed by the author for this study, and content validity was examined in two rounds of expert review. The first round served to establish the items. Both tools were reviewed by three experts holding a PhD in special education, one of whom conducts research and funded projects on disaster education for individuals with disabilities. Experts judged each item using a three-category form (appropriate, should be revised, or not appropriate) and provided written suggestions for any item not judged appropriate. No revisions were suggested for the Knowledge Level Assessment Tool, which was retained in its original form. For the Performance Assessment Tool, one expert recommended that panic responses be scored separately from calm initiation of movement toward the safe area; the scoring hierarchy was revised accordingly to include staying calm as a distinct first step (Table 3). In the second round, the revised Performance Assessment Tool was reviewed by five experts holding a PhD in special education (*n* = 3), curriculum and instruction (*n* = 1), or measurement and evaluation (*n* = 1), who rated the relevance of each item on a 4-point scale. All five experts assigned the highest rating to every item, yielding an item-level content validity index (I-CVI) of 1.00 for all items and scale-level indices of 1.00 under both the universal-agreement and averaging methods (S-CVI/UA and S-CVI/Ave; Lynn, 1986; Polit & Beck, 2006). Before data collection, the tools and procedures were piloted with one child with ASD who had characteristics similar to those of the participants and who did not take part in the study; no changes were required. The final Knowledge Level Assessment Tool consisted of 10 picture cards for pre-earthquake actions and 10 for post-earthquake actions, including 5 correct and 5 distractor items in each set to test discrimination. The complete set of tools is presented in Table 2 and Table 3.

For each item on the Knowledge Level Assessment Tool, the author presented the picture cards and asked, “Tell or show what should be done before the earthquake,” or “Tell or show what should be done after the earthquake.” Participants had a 5-s response interval. Correct responses were scored as plus, and incorrect responses or no response as minus. Performance data on the during-earthquake skill measure were scored using the 0–5 hierarchical Performance Assessment Tool during baseline probes, daily probes, post-intervention probes, generalization probes, and follow-up probes.

### 2.6. Independent Variable: Technology-Supported Behavioral Intervention Package

The independent variable was a multicomponent behavioral intervention package designed to establish stimulus control over a five-step response chain (drop–cover–hold). Each component served a distinct behavioral function: (a) a 5-min animated film produced by AFAD provided auditory–visual modeling of the antecedent stimuli (earthquake events) and the target response topographies, functioning as a discriminative stimulus (S^D^) for the safety response chain; (b) an 18-slide PowerPoint presentation containing photographs and short textual descriptions of pre-, during-, and post-earthquake actions served as a static visual reference that supported acquisition of the verbal-conceptual repertoire and supplementary stimulus control across multiple exemplars; (c) a set of visual cards used for sequencing and discrimination activities (correct vs. distractor items) served as response prompts during initial training and as conditional discrimination probes during the visual card activity; and (d) structured rehearsal of the motor response chain using a desk and within a modeled physical environment provided opportunities for in vivo response practice with contingent reinforcement.

Each instructional session lasted 27–43 min and followed the same five-phase sequence: (1) attention establishment (2 min), in which orienting responses were reinforced and the session goal was stated; (2) animated film presentation (5 min), in which the modeled stimulus-response sequence was viewed without interruption; (3) concept review with slides (10–15 min), in which the author presented each slide as an antecedent stimulus and delivered targeted comprehension questions with consequence-based feedback; (4) visual card activity (5–10 min), in which the participant arranged visual cards corresponding to pre-, during-, and post-earthquake responses, providing opportunities for conditional discrimination and response chaining; and (5) motor rehearsal (5–10 min), in which the participant performed the drop–cover–hold response chain under the discriminative stimulus (“Earthquake!”) with prompting and contingent reinforcement.

A least-to-most prompting hierarchy with response-prompt fading was used to establish stimulus control over the motor response chain: (a) independent response opportunity (5 s wait time following the discriminative stimulus), (b) verbal prompt, (c) gestural prompt, (d) partial physical prompt, and (e) full physical prompt. When an error occurred, the next level of prompt was delivered, and the trial was re-presented from the most recent successful step to maintain a high reinforcement density during acquisition. Total-task chaining, a variation of forward chaining in which the learner receives training on every step of the task analysis in each session (Cooper et al., 2020), was used to teach the five-step response sequence. In each motor rehearsal trial, the participant performed the chain from the first step (staying calm under the antecedent stimulus) to the last (holding on), and prompts were delivered at whichever step an error occurred. This format was selected because the chain is short and must be emitted as a single uninterrupted sequence in response to one antecedent event. Correct responses were followed by descriptive verbal praise functioning as a conditioned reinforcer. Token reinforcement was delivered on a continuous reinforcement schedule (CRF) during initial sessions and thinned to an intermittent variable-ratio (VR) schedule (approximately VR-3) as performance stabilized, supporting maintenance of responding under leaner schedules. Tokens were exchanged at the end of each session for participant-selected preferred items identified through a preference assessment conducted prior to baseline.

Mastery was defined as scoring 5 (maximum score) on three consecutive daily probes on the Performance Assessment Tool and 100% accuracy on the Knowledge Level Assessment Tool. The knowledge component was included because the package targeted both declarative knowledge and the motor response chain, and knowledge-level skills were taught first (Section 2.11); the criterion was intended to ensure that both components had been acquired before a participant proceeded. All three participants met this component, with posttest scores of 100% on both item sets (Section 3.1); this component was assessed at the posttest, which was conducted immediately after the performance component had been met (Section 2.8). Because knowledge was not measured repeatedly within the design, this component of the criterion does not contribute to the demonstration of experimental control. Participants who met the criterion proceeded to the next probe condition and to the post-instruction generalization sessions (Section 2.9 and Section 2.12). Sessions were conducted three times per week in a one-to-one instructional arrangement with the author. The intervention phase comprised 6 sessions for Utku (sessions 4–9), 6 for Kaan (sessions 13–18), and 7 for Atakan (sessions 22–28). The content of the intervention package was developed through a review of prior earthquake preparedness research and refined based on recommendations from three doctoral-level experts in special education.

### 2.7. Experimental Procedure

The experimental procedure consisted of pretest and posttest sessions, baseline probes, daily probe sessions, instructional sessions, generalization probes, and follow-up sessions. All sessions were conducted as one-to-one instructional arrangements. The response interval was 5 s across all sessions. During instructional sessions, correct responses were followed by descriptive verbal praise and token reinforcement, with tokens exchanged for preferred items (e.g., toy cars, soccer cards, children’s magazines, crayons), as described in Section 2.6. During probe sessions (baseline, daily, and post-intervention), generalization sessions, and follow-up sessions, correct and incorrect responses received no programmed consequences; participation was acknowledged with general verbal praise once the probe had ended, as described in Section 2.9. Instructional sessions continued until the mastery criterion was met (Section 2.6).

### 2.8. Pretest and Posttest Sessions

Pretest scores on the Knowledge Level Assessment Tool established baseline performance prior to intervention. Posttest scores collected immediately following the intervention phase served as the measure of knowledge acquisition. Pretest and posttest sessions were conducted individually with each participant in the instructional classroom. During these sessions, the author presented the assessment items and recorded responses on the data collection form. No prompts, reinforcement, or error correction were delivered during pretest or posttest sessions.

### 2.9. Probe-Condition Schedule

Probe conditions (bulk probes, labeled BP in Figure 1) were conducted at four time points across the study following the probe-condition multiple-probe design (Horner & Baer, 1978; Ledford & Gast, 2018). Each probe condition consisted of three consecutive assessment sessions in which performance on the during-earthquake skill measure was assessed under strict test conditions. During probe sessions, no prompts (verbal, gestural, modeling, or physical) were delivered, no programmed consequences followed correct or incorrect responses, and no corrective feedback was given. Each probe session consisted of a single trial: the discriminative stimulus (“Earthquake!”) was presented once, and the participant’s response was scored on the 0–5 rubric without any researcher intervention. To prevent inhibitive testing effects associated with repeated assessment in the absence of reinforcement, participation was reinforced with general verbal praise (e.g., “you’re great,” “you did a great job”) delivered once, after the trial and before the participant left the session, independent of response accuracy (Ledford & Gast, 2018); praise was therefore delivered in every probe session, including baseline probes in which all responses were scored 0. No tangible, token, or activity reinforcers were delivered in probe sessions. This procedural separation was implemented to ensure that probe data reflected true levels of independent responding rather than prompted or reinforced performance, consistent with single-case methodological standards for probe-condition multiple-probe designs (Ledford & Gast, 2018). Probe 1 served as the baseline probe condition for all three participants and was conducted with all three participants in sessions 1–3. The same procedures were applied in all four probe conditions, in the daily probes conducted during the intervention phase (Section 2.10), and in generalization and follow-up sessions (Section 2.12).

The intervention was then introduced to the three participants in a time-lagged (staggered) sequence. Participant 1 (Utku) entered the intervention phase at session 4, immediately following Probe 1, while Participants 2 (Kaan) and 3 (Atakan) remained in the baseline condition and were assessed in the subsequent probe condition. Probe 2 was conducted across all three participants at sessions 10–12, after Participant 1 had reached the mastery criterion. Participant 2 entered the intervention phase at session 13, immediately following Probe 2, while Participant 3 remained in the baseline condition. Probe 3 was conducted across all three participants at sessions 19–21, after Participant 2 had reached the mastery criterion. Participant 3 entered the intervention phase at session 22, immediately following Probe 3. Probe 4 was conducted across all three participants at sessions 29–31, after Participant 3 had reached the mastery criterion. This staggered introduction across tiers, with the entry of each successive participant contingent on the preceding participant having reached the mastery criterion, allowed evaluation of whether performance changed only upon introduction of the intervention for that participant, providing the basis for inferring a functional relation. Three within-study replications of the predicted effect were anticipated, one for each participant.

The full schedule was therefore as follows: Sessions 1–3 (Probe 1: all participants in baseline); Sessions 4–9 (intervention for Participant 1; Participants 2 and 3 remained in baseline and were not assessed); Sessions 10–12 (Probe 2: all participants); Sessions 13–18 (intervention for Participant 2; Participant 1, who had met the mastery criterion, received no instruction and was assessed only in scheduled follow-up probes, and Participant 3, who remained in baseline, received neither instruction nor assessment); Sessions 19–21 (Probe 3: all participants); Sessions 22–28 (intervention for Participant 3; Participants 1 and 2, who had met the mastery criterion, received no instruction and were assessed only in scheduled follow-up probes); Sessions 29–31 (Probe 4: all participants). Follow-up probes were conducted at 2, 4, and 8 weeks after each participant’s own last instructional session. This schedule is summarized session by session in Table 4 and depicted in Figure 1.

### 2.10. Daily Probe Sessions

Daily probe sessions were conducted at the beginning of each instructional session, before any instruction was delivered, throughout the intervention phase. Each daily probe therefore assessed responding after a delay from the preceding instructional session rather than immediately after instruction, so that the data reflected retained, independent performance rather than short-term recency effects. Because the first daily probe was conducted at the start of the first instructional session, it preceded all instruction; each subsequent daily probe reflected performance after one additional completed instructional session. Daily probe scores constitute the intervention-phase data in Figure 1. Daily probes were conducted under conditions identical to the probe conditions (Section 2.9): the discriminative stimulus (“Earthquake!”) was presented, the participant was given a 5-s response interval, no prompts of any kind were delivered, and no programmed consequences followed correct or incorrect responses. Participation was acknowledged with general verbal praise once the probe had ended, independent of response accuracy. Instruction, prompting, and contingent reinforcement of correct responding occurred only in the instructional portion of the session that followed (Section 2.11). Daily probes continued until the participant met the performance component of the mastery criterion (a score of 5 on three consecutive daily probes; Section 2.6).

### 2.11. Instructional Sessions

Instructional sessions used the technology-supported package described in Section 2.6. Knowledge-level skills were taught first, followed by during-earthquake motor skills (staying calm, identifying the safe area, collapsing, closing, and holding on). Direct instruction was used as the primary instructional method, supported by the technology-based components.

### 2.12. Generalization and Follow-Up Sessions

Follow-up sessions were conducted at 2, 4, and 8 weeks after each participant’s own last instructional session. Generalization sessions were structured similarly to probe sessions. They incorporated novel materials (10 × 10 cm cardboard picture cards with changed size, font, typeface, and colors), a novel setting (university foyer), and a novel implementer (a second special education specialist). The three dimensions were changed together in every generalization session, so that each generalization probe assessed responding under a combination of novel setting, materials, and implementer. Generalization sessions were conducted after Probe 1 (pre-intervention), after the participant’s instructional sessions were completed (post-intervention), and at each follow-up point (2, 4, and 8 weeks). The response interval was 5 s, and the inter-trial interval between knowledge items was 5 s. Three generalization sessions were conducted at each of these time points, and the mean of the three sessions is reported in Section 3.4. In both generalization and follow-up sessions, consequences were identical to those in probe sessions: responses received no programmed consequences, and participation was acknowledged with general verbal praise at the end of the session, independent of response accuracy (Section 2.9).

### 2.13. Interobserver Agreement and Treatment Integrity

Prior to data collection, an independent observer holding a PhD in special education was trained on the data collection protocol and intervention procedures, following recommended practice for observer training (Ledford & Gast, 2018). The observer was given the Performance Assessment Tool and the operational definition of each step, including the definition of staying calm (Section 2.5); the author and the observer then scored practice trials together, discussed disagreements, and independently scored further trials against an a priori criterion of at least 80% point-by-point agreement. Agreement on the final calibration trials was 100%, and calibration was completed before the pilot study. Interobserver agreement (IOA) data were collected for 30% of baseline, intervention, and follow-up sessions, and 50% of generalization sessions for all participants, using video recordings of randomly selected sessions. IOA was calculated using the point-by-point agreement method: IOA = (agreements/[agreements + disagreements]) × 100. Mean IOA across all phases was 94% (range: 90–100%). Phase-by-phase mean IOA was as follows: baseline (*M* = 96%, range: 92–100%), intervention (*M* = 93%, range: 90–100%), generalization (*M* = 95%, range: 90–100%), and follow-up (*M* = 94%, range: 92–100%).

Treatment integrity was assessed using a procedural checklist that enumerated the steps for each session component. The independent observer scored the percentage of correctly implemented intervention steps from video recordings of randomly selected sessions, with 30% of intervention sessions assessed for each participant. Treatment integrity = (steps completed correctly/total steps) × 100. Mean treatment integrity was 99% (range: 98–100%) across all assessed sessions and participants.

### 2.14. Social Validity Assessment

Social validity data were collected using semi-structured interview questions reviewed and approved by three experts in the field before use; the questions were also approved by the ethics committee as part of the study application. Interviews were conducted with the participants and their mothers following completion of the intervention. The interview with mothers comprised five questions: (1) What do you think about this training on earthquake protection skills provided to your child? (2) What do you think about the changes in your child’s behavior after the training? (3) What do you think about other children with special needs or typically developing peers receiving this training? (4) What other topics would you like a training on earthquake protection skills to include? (5) What did you like and dislike about the study? Participants were asked two simplified yes/no questions: whether the training was useful and whether they would like similar training again. Mothers also reported on observed changes in their children’s behavior, including naming earthquake-related actions and preparing an earthquake bag at home. Themes were identified by the author through descriptive thematic analysis of the interview responses; no second coder was involved.

### 2.15. Data Analysis

Analysis and reporting of the primary dependent variable (skill-level performance) followed What Works Clearinghouse (WWC) standards for single-case designs (Kratochwill et al., 2010; What Works Clearinghouse, 2020) and the Single-Case Reporting Guideline in Behavioural Interventions (SCRIBE; Tate et al., 2016). Data were plotted in line graphs (Figure 1) using the 0–5 raw score from the Performance Assessment Tool, and each participant’s data were visually inspected across five dimensions: level, trend, immediacy of effect, variability, and overlap between baseline and intervention phases (Kratochwill et al., 2010; Ledford & Gast, 2018). To supplement visual analysis with a quantitative index of effect, Tau-U effect sizes were calculated for each participant using the web-based application developed by Vannest et al. (2011), which also yields 90% confidence intervals (CIs). Tau-U was selected because it quantifies nonoverlap between phases and permits correction for baseline trend when such a trend is present. Tau-U was calculated by comparing each participant’s baseline probe-condition data (Probe 1 for Utku; Probes 1–2 for Kaan; Probes 1–3 for Atakan) with all subsequent probe data for that participant, namely the daily probes of the intervention phase and the post-intervention probe conditions; follow-up probes were not included. Each baseline–intervention data pair was classified as improving, deteriorating, or tied; tied pairs contributed zero to the numerator and were retained in the denominator, so that ties lower the index rather than being discarded. Because visual inspection confirmed flat, stable, and zero-trend baseline data across all three participants, the unadjusted Tau-U (Tau-U A) was reported without correction for baseline trend, in accordance with recommended practice when baseline data show no systematic trend (Parker et al., 2011; Vannest & Ninci, 2015/2016). Effect sizes were interpreted using the guidelines proposed by Vannest and Ninci (2015/2016), under which values up to 0.20 indicate a small change, 0.20 to 0.60 a moderate change, 0.60 to 0.80 a large change, and above 0.80 a large to very large change. Because overlap-based indices capture only one dimension of single-case data and are sensitive to the number of data points in each phase, Tau-U was treated as a supplement to visual analysis rather than as an independent estimate of effect magnitude (Ledford & Gast, 2018). Knowledge-level data, treated as a secondary descriptive measure, were summarized using descriptive statistics in Table 5 and Table 6, and skill-level generalization data in Table 7. Social validity data were analyzed using descriptive thematic analysis of interview responses.

## 3. Results

Results are presented in two parts. First, descriptive results for knowledge-level acquisition and generalization are reported as supplementary descriptive results characterizing knowledge change. Second, the primary single-case experimental results for skill-level performance are reported with visual analysis and Tau-U effect sizes. Tau-U values are interpreted using the guidelines stated in Section 2.15.

### 3.1. Knowledge-Level Acquisition and Retention (Descriptive)

Table 5 presents knowledge-level performance across phases. Because knowledge-level data were collected as a discrete pretest-posttest-follow-up measure rather than through repeated probes within the multiple-probe design, these results are reported descriptively and do not provide a basis for inference about experimental control. All three participants increased from pretest levels (0–10%) to 100% accuracy at posttest on both pre-earthquake and post-earthquake action knowledge. Performance was maintained at 100% across all three follow-up probes (2, 4, and 8 weeks post-intervention) for all participants on both knowledge components.

### 3.2. Knowledge-Level Generalization Across Settings, Persons, and Materials (Descriptive)

Table 6 presents knowledge-level generalization performance across novel settings (university foyer), novel materials (different-sized cards with altered font and color), and a novel implementer (a second special education specialist). All three participants showed pre-intervention generalization performance of 0–10% on both pre- and post-earthquake action knowledge components. Following the intervention, all participants achieved 100% accuracy on generalization probes and maintained this level across all three follow-up phases.

### 3.3. Skill-Level Acquisition and Maintenance (Primary Outcome)

Visual analysis of Figure 1 indicated a functional relation between the behavioral intervention package and acquisition of the earthquake safety response chain, with three within-study replications of effect across participants. As set out in the Limitations, the three baseline probe-condition data points obtained for the first tier (Utku) place the design in the “Meets Standards With Reservations” category under What Works Clearinghouse (2020) Version 4.1, and the demonstration is therefore reported as replicated but provisional. Across all three participants, baseline probe-condition data demonstrated stable responding at zero level, zero variability, and zero trend across the entire baseline phase. In most baseline probe sessions the score of 0 reflected competing responses (e.g., running, screaming, moving toward the window); in one or two sessions a participant remained motionless and did not initiate movement toward the safe area, which was also scored 0 under the definition of Step 1 (Section 2.5). Following the introduction of the intervention, a change in level was observed for each participant, with a rising trend culminating in performance at the maximum level of the response measure. Because each daily probe preceded that day’s instruction, the first intervention-phase data point represents performance before any instruction. The change was rapid for Utku and Kaan, who scored 0 before instruction and after the first instructional session, 3 after the second, and 5 after the third, and more gradual for Atakan, who passed through intermediate scores (2 and 3) and first scored 5 after the fourth instructional session. All three participants then met the mastery criterion of three consecutive scores of 5. No overlap was observed between baseline probe-condition data and post-intervention probe-condition data for any participant, demonstrating complete separation between baseline and post-intervention probe-condition data. Tau-U effect size values, calculated by comparing baseline probe-condition data (all probe conditions preceding intervention for each participant) with all daily probe data from the intervention phase and post-intervention probe-condition data, were 0.87 (90% CI [0.25, 1.00], *p* = 0.021) for Utku, 0.83 (90% CI [0.35, 1.00], *p* = 0.005) for Kaan, and 0.80 (90% CI [0.35, 1.00], *p* = 0.003) for Atakan. Under the guidelines of Vannest and Ninci (2015/2016), the values for Utku and Kaan indicate a large to very large change and the value for Atakan a large change. Values below 1.00 arise solely from the two intervention-phase scores of 0 for each participant (the probe preceding the first instructional session and the probe following it), which are tied with the baseline scores; every other intervention-phase and post-intervention score exceeded every baseline score.

For Utku, baseline probe-condition data (Probe 1, sessions 1–3) were stable at score = 0 (*M* = 0.00, range = 0–0). The intervention was introduced at session 4. The daily probe preceding the first instructional session yielded a score of 0, as did the probe following the first instructional session; the score rose to 3 after the second instructional session and reached the maximum score (5) after the third, which was maintained across the next two daily probes, meeting the mastery criterion. Across the six daily probes, the mean score was 3.00 (range = 0–5). Post-intervention probe conditions (Probes 2–4, sessions 10–12, 19–21, and 29–31) were stable at score = 5 across all nine probe sessions (*M* = 5.00), indicating maintenance of responding under the same conditions as the baseline probes, in which responses received no programmed consequences. Within the intervention phase, Utku’s data showed low variability once the maximum score was reached, suggesting rapid acquisition and stable responding once the response chain was established. At follow-up, Utku scored 5 at the 2-week and 8-week probes; the 4-week probe yielded a score of 4, reflecting absence of the terminal response (holding on) at that single probe.

For Kaan, baseline probe-condition data (Probe 1, sessions 1–3, and Probe 2, sessions 10–12) were stable at score = 0 (*M* = 0.00, range = 0–0) across six probe sessions. The continued zero responding during Probe 2, while Utku had already reached the mastery criterion, together with Kaan’s subsequent change after the intervention was introduced, constitutes the second replication of effect and is consistent with the behavior change being contingent on the introduction of the intervention rather than on the passage of time. The intervention was introduced at session 13. As for Utku, the daily probes preceding and following the first instructional session yielded scores of 0; the score rose to 3 after the second instructional session and reached 5 after the third, which was maintained across the next two daily probes, meeting the mastery criterion. Across the six daily probes, the mean score was 3.00 (range = 0–5). Subsequent post-intervention probe conditions (Probes 3–4, sessions 19–21 and 29–31; *M* = 5.00) and follow-up probes at 2, 4, and 8 weeks were all stable at score = 5.

For Atakan, baseline probe-condition data (Probes 1–3, sessions 1–3, 10–12, and 19–21) were stable at score = 0 (*M* = 0.00, range = 0–0) across nine probe sessions. The continued zero responding during Probes 2 and 3, while Utku and Kaan had already reached the mastery criterion, together with Atakan’s subsequent change after the intervention was introduced, constitutes the third replication of effect. The intervention was introduced at session 22. Atakan showed a more gradual acquisition pattern than the other two participants: the daily probes preceding and following the first instructional session yielded scores of 0, scores rose to 2 and 3 after the second and third instructional sessions, and reached 5 after the fourth, which was maintained across the next two daily probes, meeting the mastery criterion. Across the seven daily probes, the mean score was 2.86 (range = 0–5). Variability within the intervention phase was somewhat higher for Atakan during acquisition (scores of 0, 0, 2, and 3 across the first four daily probes); once the maximum score was reached, responding was stable with no within-phase variability. The subsequent post-intervention probe condition (Probe 4, sessions 29–31; *M* = 5.00) and follow-up probes at 2, 4, and 8 weeks were all stable at score = 5.

### 3.4. Skill-Level Generalization

Table 7 presents skill-level performance across novel settings, materials, and implementer conditions. All three participants obtained a mean score of 0 on the during-earthquake skill measure in the pre-intervention generalization sessions. Following the intervention, all participants obtained a mean score of 5 under generalization conditions; at follow-up, mean scores remained at 5 except for Utku’s mean score of 4 at 4 weeks.

### 3.5. Social Validity

Semi-structured interviews with the three mothers and the three participants yielded descriptive data organized around three themes identified through analysis of interview responses. The first theme, perceived necessity, reflected mothers’ reports that earthquake safety instruction tailored to children with ASD addressed a need not previously met by general school-based safety education. As one mother put it, “As people who have recently lived through an earthquake, it is very valuable for our children to acquire these skills”; another said, “We do not know when an earthquake will happen; it is important to me that he knows what to do if it happens at school or anywhere else when we are not with him.” The second theme, observed behavioral change at home, included reports from all three mothers that, following the intervention, their children initiated discussions about earthquake-related actions and participated in preparing an earthquake bag at home. The third theme, acceptability and recommendation, reflected mothers’ indication that they would recommend similar instruction for other children with special needs and for typically developing children. One mother also described her son’s engagement: “He came to the sessions gladly and willingly; he enjoyed working with you.” Interview responses were given in Turkish and were translated by the author. Participants’ own ratings on simplified yes/no questions about whether the training was useful and whether they would want similar training again were uniformly affirmative; these responses are reported as descriptive feedback rather than as evidence of social validity. These descriptive social validity findings should be interpreted with caution, given the limitations of self-report data collected from a small group of stakeholders directly involved in the study.

## 4. Discussion

The present study evaluated a technology-supported instructional package designed to teach earthquake safety skills to three children with ASD using a probe-condition multiple-probe across-participants single-case experimental design. Findings provide preliminary evidence that the package was associated with increases in skill-level performance from baseline (score = 0) to mastery (score = 5) for all three participants, with the criterion score maintained across post-intervention probe conditions and follow-up probes at 2, 4, and 8 weeks, except for a single score of 4 for Utku at 4 weeks. Skill performance also generalized across a novel setting, novel materials, and a novel implementer. Tau-U effect sizes for the primary skill-level outcome ranged from 0.80 to 0.87 across the three participants, with all 90% confidence intervals excluding zero and all *p*-values below the conventional 0.05 threshold; under the guidelines of Vannest and Ninci (2015/2016), these values indicate a large (Atakan) to large-to-very-large (Utku and Kaan) change. As the values fall below 1.00 solely because of tied zero scores at the start of the intervention phase, and because Tau-U captures a single dimension of the data, they are interpreted as supplementing rather than replacing the visual analysis. Visual analysis was consistent with a functional relation across three within-study replications: each participant’s data changed only after the introduction of the intervention, while non-intervention participants remained at baseline. Because the first tier (Utku) had three rather than five baseline probe-condition data points, it meets What Works Clearinghouse standards with reservations; the demonstration of experimental control should accordingly be treated as provisional and in need of replication rather than as established. These findings should be interpreted within the constraints of a small-sample single-case experimental design and require systematic replication before broader conclusions can be drawn.

The present findings extend prior work in several ways. First, whereas Kurt et al. (2024) evaluated drop–cover–hold responses using video modeling, the present study established stimulus control over a five-step behavioral chain through a multicomponent package combining video modeling, conditional discrimination training, and in vivo response rehearsal with least-to-most prompt fading. Both studies observed acquisition and short-term maintenance, suggesting that systematic behavioral instruction can establish earthquake safety responding across different procedural arrangements. The same conclusion is supported by Ulaşman and Sivrikaya (2025), who used digital social stories with primary school students, and by Gürsoy and Kot (2026), who used video modeling with adolescents; with the present study, four single-case evaluations using different procedures, participants, and settings have now reported the acquisition of earthquake safety responses by learners with ASD. Second, the present study extended follow-up to 8 weeks, providing initial evidence of medium-term maintenance in the absence of programmed consequences for responding. The temporary decrement observed for one participant (Utku) at the 4-week probe, in which the holding-on response (the terminal step in the five-step chain) was absent while the four preceding steps were performed, warrants behavioral interpretation. The dip was isolated to the terminal step rather than reflecting general response decay, which is consistent with two complementary behavioral explanations. First, the terminal step was among the last to be performed independently during acquisition (Section 3.3), so it had a shorter history of reinforced independent performance than the earlier steps before the reinforcement schedule was thinned and may therefore have been more vulnerable to extinction effects. Second, holding on requires sustained motor responding under a static stimulus condition (post-collapse), a configuration in which the discriminative stimulus for continued responding is less salient than the cues for the earlier steps. Both interpretations support the inclusion of booster sessions specifically targeting terminal chain components and the use of fading procedures that retain occasional reinforcement of the full chain rather than only its individual components. The importance of carefully designed fading procedures in maintaining behavioral gains has been illustrated in the broader instructional literature for learners with developmental disabilities; for instance, Brady-Ruehs et al. (2024) compared two procedures for fading video supports and found that, although both improved independent performance of daily living tasks, posttest performance was higher for the skills taught with the procedure that faded to video models. These interpretations are offered as hypotheses for future research rather than as evidence about the mechanisms of the present package.

Although rapid acquisition was observed across participants, the present design does not permit isolation of technology components as the sole contributing factor. Other variables, including intensive one-to-one instruction, a controlled setting free of competing stimuli, and a dense schedule of reinforcement, may also have contributed to the observed outcomes. Future component analyses would be required to disentangle the contributions of specific instructional features. The temporary decrease at 4-week follow-up may reflect typical retention patterns observed in skill-acquisition research rather than a characteristic specific to ASD; this finding underscores the value of incorporating planned booster sessions to strengthen long-term maintenance, particularly for safety-critical skills.

Generalization to the novel setting (university foyer), materials, and implementer suggests transfer of trained behaviors beyond the original instructional conditions. Because the three dimensions were changed together, the results show that responding occurred under this combination of novel conditions; they do not show which dimension, if any, would have disrupted responding had it been varied alone. In addition, generalization was assessed before instruction, after instruction, and at follow-up rather than repeatedly within the design, so it was not subject to experimental control (Ledford & Gast, 2018). Nor does this finding constitute evidence of real-world safety effectiveness during an actual earthquake event. Ecological validity remains a substantial limitation, as performance was evaluated under controlled instructional conditions rather than during a seismic event. Future research should evaluate performance under simulated conditions that more closely approximate the sensory and emotional features of an earthquake. This is particularly important for autistic children, in whom sensory processing differences are associated with heightened neurophysiological stress and anxiety (Pergantis et al., 2025): sirens, shaking, crowding, and uncertainty may disrupt a response chain that was mastered in a quiet room. Training could therefore be extended by graded exposure to such features during rehearsal, and the emerging technologies reviewed by Pergantis et al. (2025) for detecting and reducing stress may help in planning and monitoring that adaptation. This literature bears on the sensory context and future adaptation of training; it does not provide evidence for the effectiveness of the present package.

A methodological note concerns the measurement of declarative knowledge in this study. Knowledge-level performance was assessed at pretest, posttest, and follow-up rather than through repeated probes within the multiple-probe design. This decision reflected two considerations. First, repeated administration of the same set of knowledge items across sessions would introduce a testing effect: rehearsal of the assessment items themselves could function as a form of distributed practice, thereby confounding the effects of the instructional package with the effects of repeated test administration. Second, declarative knowledge of safety actions was a secondary target that complemented the motor response chain, whereas the primary dependent variable for inference about a functional relation was the overt motor response, which was repeatedly probed under the multiple-probe design. Knowledge-level data are therefore reported as descriptive supplementary information and are not interpreted as providing experimental control.

### 4.1. Practical and Policy Implications

These preliminary findings have implications for the development of disability-inclusive disaster preparedness protocols and add to a small but growing literature on structured behavioral interventions that teach safety-relevant repertoires to children with ASD. Social validity findings, organized around themes of perceived necessity, observed behavioral change at home, and acceptability, suggest that the intervention was acceptable to families, and the home behaviors reported by mothers (e.g., preparing an earthquake bag) provide an indirect indication of intervention effects in non-instructional settings. Following systematic replication with larger, more diverse samples, the integration of similar packages into special education curricula could be explored as a long-term direction. The present study does not provide sufficient evidence to support immediate widespread adoption or national curricular integration; such broader applications should be conditioned on replication, component analysis, and evaluation under more ecologically valid conditions.

### 4.2. Limitations

Three categories of limitations should be considered. First, sample and design limitations: the small sample size (*N* = 3) is inherent to single-case experimental designs and limits the generalizability of findings. Systematic replication across diverse participants, settings, and implementers is required before broader conclusions can be drawn. The sample also included only male participants in a single geographic context. A further design consideration involves the number of baseline probe-condition data points obtained prior to intervention. Under the What Works Clearinghouse (2020) Version 4.1 standards for single-case designs, phases containing five or more data points are eligible for the “Meets Standards Without Reservations” classification, whereas phases with three or four data points are eligible for the “Meets Standards With Reservations” classification, provided that the data are stable. Consistent with common practice in probe-condition multiple-probe designs, each probe condition in the present study comprised three sessions, so that the first tier (Utku) had three baseline probe-condition data points prior to intervention and the second and third tiers had six and nine; all baseline data points were stable at a zero level with zero trend and zero variability across participants (Ledford & Gast, 2018). Under WWC 4.1, this design would therefore be classified as “Meets Standards With Reservations,” a limitation that should be considered when interpreting the strength of the demonstration of experimental control. Future replications may strengthen the design classification by extending baseline probe assessments to five or more probe-condition data points per tier where feasible. Second, measurement limitations: ecological validity is constrained because the study evaluated performance under controlled instructional conditions rather than during an actual seismic event or community-based drill. In addition, both assessment tools were developed for this study; their content validity was supported by two rounds of expert review and their scoring consistency by interobserver agreement, but their construct validity and test–retest reliability were not examined. The Performance Assessment Tool also assigns a score of 0 both when a response incompatible with safety occurs and when the participant does not initiate the protective sequence; future studies should record these two response classes separately. Third, follow-up duration: maintenance probes extended to 8 weeks post-intervention, after which the data collection was discontinued because of the school summer break. Future studies should incorporate a minimum 12-week follow-up to assess longer-term retention of these life-relevant skills.

A further methodological limitation concerns the measurement of social validity. Social validity in the present study was evaluated through semi-structured interviews with mothers and participants and analyzed thematically. Although this qualitative approach provided rich contextual information, single-case and applied behavior-analytic journals have traditionally favored standardized quantitative social validity instruments (e.g., Likert-type acceptability scales; Kazdin, 1982; Wolf, 1978) that yield interpretable scores comparable across studies. Future replications should supplement qualitative interviews with a standardized quantitative social validity measure to strengthen inferences regarding the acceptability and feasibility of the instructional package for stakeholders. In addition, the interview themes were identified by a single coder, without an independent check of the coding.

### 4.3. Future Research

Three priority directions are suggested. First, systematic replication with larger and more diverse samples (including female participants, broader age ranges, and varied severity profiles) across multiple settings and implementers is needed to establish the generalizability of the present findings. Second, evaluation of booster session protocols and extended follow-up periods of at least 12 weeks would clarify long-term maintenance and inform recommendations for spaced practice. Third, ecological validity could be strengthened by evaluating performance under simulation conditions, scheduled disaster drills, or in real-world settings such as participants’ homes and schools.

## 5. Conclusions

This single-case experimental study offers preliminary evidence that a technology-supported, multicomponent behavioral intervention can support the acquisition of earthquake safety skills in children with ASD, and that the acquired responding was maintained for up to 8 weeks and occurred under the untrained conditions sampled here. The maintenance and generalization findings were obtained under controlled conditions with a limited set of novel settings, materials, and implementers, and one participant showed a temporary decrement at the 4-week follow-up; they should therefore be read as evidence of persistence and transfer within those conditions rather than as evidence of durable performance during an actual seismic event. The results add to the systematic-instruction literature on safety skills in this population and speak to the value of an integrated package that targets both declarative knowledge and overt motor responses. Because the package was evaluated as a whole, the study does not show which component produced the change, and the contribution of the technology-based materials cannot be separated from that of direct instruction, prompting, rehearsal, and reinforcement. Systematic replication with larger and more diverse samples is needed before the findings can be extended to broader instructional practice.

## Figures and Tables

**Table 1:** Participant Characteristics.

Participant	Age	ASD Diagnosis	Diagnostic Tool	Prerequisite Skills	Educational Setting
Utku	8y 7m	Clinical	DSM-5	Met	Special Education Class
Kaan	10y 2m	Clinical	DSM-5	Met	Inclusive
Atakan	12y 7m	Clinical	DSM-5	Met	Inclusive

Note. DSM-5 = Diagnostic and Statistical Manual of Mental Disorders (5th ed.). Pseudonyms are used to protect participant identity.

**Table 2:** Knowledge Level Assessment Tool: Pre-Earthquake Items.

Item	Action Shown on the Card	Correct	Distractor
1	Preparing an earthquake bag	✓	
2	Preparing a sports bag		✓
3	Fixing items in the house	✓	
4	Sleeping in bed		✓
5	Learning the location of electricity and natural gas valves	✓	
6	Washing hands at a fountain		✓
7	Knowing emergency telephone numbers	✓	
8	Taking the elevator		✓
9	Knowing emergency exit and evacuation routes	✓	
10	Going to the accessible toilet		✓

Note. The Post-Earthquake set followed the same 5-correct/5-distractor structure. ✓ indicates the category of each item (correct action or distractor).

**Table 3:** Performance Assessment Tool: Scoring System for During-Earthquake Skills.

Score	Behavioral Indicator
5	Holds on (in addition to all behaviors below)
4	Closes into fetal position (in addition to all behaviors below)
3	Collapses to the ground (in addition to all behaviors below)
2	Identifies and remains within the safe area (in addition to staying calm)
1	Stays calm and initiates movement toward the safe area
0	Does not meet Step 1: displays behaviors incompatible with safety (e.g., panicking, running suddenly, screaming, moving toward a window, exit door, stairs, or elevator) or does not initiate movement toward the safe area

Note. The scoring system is hierarchical and cumulative; a score of 5 indicates performance of all five target behaviors. The operational definition applied when scoring Step 1 (stays calm) is reported in Section 2.5.

**Table 4:** Session-by-session schedule of assessment and instruction for each participant.

Sessions	Utku	Kaan	Atakan
1–3	Probe 1 (baseline)	Probe 1 (baseline)	Probe 1 (baseline)
4–9	Instruction; daily probe at the start of each session (6 probes)	Baseline; not assessed	Baseline; not assessed
10–12	Probe 2 (post-intervention)	Probe 2 (baseline)	Probe 2 (baseline)
13–18	Mastery criterion met; no instruction; assessed only in scheduled follow-up probes	Instruction; daily probe at the start of each session (6 probes)	Baseline; not assessed
19–21	Probe 3 (post-intervention)	Probe 3 (post-intervention)	Probe 3 (baseline)
22–28	No instruction; assessed only in scheduled follow-up probes	No instruction; assessed only in scheduled follow-up probes	Instruction; daily probe at the start of each session (7 probes)
29–31	Probe 4 (post-intervention)	Probe 4 (post-intervention)	Probe 4 (post-intervention)
Follow-up	Probes at 2, 4, and 8 weeks after the participant’s last instructional session	Probes at 2, 4, and 8 weeks after the participant’s last instructional session	Probes at 2, 4, and 8 weeks after the participant’s last instructional session

Note. In all probe conditions, daily probes, and follow-up probes, responses received no programmed consequences, and participation was acknowledged with general verbal praise at the end of the probe; prompting and contingent reinforcement of correct responses occurred only during instruction (Section 2.9, Section 2.10, Section 2.11 and Section 2.12). Each daily probe was conducted at the start of an instructional session, before instruction. Follow-up probes were timed from each participant’s own last instructional session and therefore, for Utku and Kaan, took place during the later phases of the study; they are listed in the final row and plotted at the end of Figure 1. Generalization sessions (Section 2.12) and knowledge-level pretest and posttest sessions (Section 2.8) are not shown.

**Table 5:** Knowledge-Level Performance Across Phases (Percentage of Correct Responses).

Participant	Component	Pretest	Posttest	Follow-Up 1 (2 wk)	Follow-Up 2 (4 wk)	Follow-Up 3 (8 wk)
Utku	Pre-earthquake	10	100	100	100	100
Utku	Post-earthquake	10	100	100	100	100
Kaan	Pre-earthquake	10	100	100	100	100
Kaan	Post-earthquake	10	100	100	100	100
Atakan	Pre-earthquake	0	100	100	100	100
Atakan	Post-earthquake	0	100	100	100	100

Note. All values represent the percentage of correct responses on the Knowledge Level Assessment Tool. Follow-up probes were conducted at 2, 4, and 8 weeks after each participant’s own last instructional session.

**Table 6:** Knowledge-Level Generalization Across Novel Settings, Materials, and Implementers.

Participant	Component	Pre-Intervention	Post-Intervention	FU 1 (2 wk)	FU 2 (4 wk)	FU 3 (8 wk)
Utku	Pre-earthquake	10	100	100	100	100
Utku	Post-earthquake	10	100	100	100	100
Kaan	Pre-earthquake	10	100	100	100	100
Kaan	Post-earthquake	10	100	100	100	100
Atakan	Pre-earthquake	0	100	100	100	100
Atakan	Post-earthquake	0	100	100	100	100

Note. Generalization probes were conducted in a novel setting (university foyer), with novel materials (10 × 10 cm cards with altered font and color), and with a novel implementer (a second special education specialist). FU = Follow-up. Values are means of three generalization sessions at each time point.

**Table 7:** Skill-Level Performance Across Novel Settings, Materials, and Implementer (Raw Scores on the 0–5 Performance Assessment Tool).

Participant	Pre-Intervention	Post-Intervention	FU 1 (2 wk)	FU 2 (4 wk)	FU 3 (8 wk)
Utku	0	5	5	4	5
Kaan	0	5	5	5	5
Atakan	0	5	5	5	5

Note. Values represent the mean of three generalization sessions at each time point, in raw scores on the hierarchical 0–5 Performance Assessment Tool, where 5 indicates cumulative performance of all five target safety behaviors, and 0 indicates that Step 1 was not met (Table 3). Generalization conditions included a novel setting (university foyer), novel materials, and a novel implementer. FU = Follow-up. Utku’s mean score of 4 at the 4-week follow-up represents a decrement from the maximum score.

## Data Availability

The aggregated performance data supporting the findings of this study are presented within the article (Table 1, Table 2, Table 3, Table 4, Table 5, Table 6 and Table 7, Figure 1). The underlying raw session data, including video recordings and individual scoring protocols, are not publicly available because they contain identifiable information about minor participants with autism spectrum disorder; these data can be made available by the corresponding author upon reasonable request, subject to compliance with the ethics committee approval and the terms of parental informed consent.

## References

[B1-behavsci-16-01897] Alexander J. L., Ayres K. M., Shepley S. B., Smith K. A., Ledford J. R. (2017). Comparison of probe procedures in the assessment of chained tasks. The Psychological Record.

[B2-behavsci-16-01897] Alghamdi M., Alhakbani N., Al-Nafjan A. (2023). Assessing the potential of robotics technology for enhancing educational for children with autism spectrum disorder. Behavioral Sciences.

[B3-behavsci-16-01897] American Psychiatric Association (2022). Diagnostic and statistical manual of mental disorders.

[B4-behavsci-16-01897] Brady-Ruehs A. M., Carreon A., Van Laarhoven T., Johnson J., Chandler L. (2024). Comparing the effectiveness of two video fading procedures for teaching students with developmental disabilities daily living skills. Education and Treatment of Children.

[B5-behavsci-16-01897] Centre for Research on the Epidemiology of Disasters (CRED) (2024). EM-DAT: The international disaster database.

[B6-behavsci-16-01897] Cooper J. O., Heron T. E., Heward W. L. (2020). Applied behavior analysis.

[B7-behavsci-16-01897] Eroğlu M., Yakşi N. (2023). Changes in symptom severity and sleeping habits after earthquake in children and adolescents with autism spectrum disorder: The case of the Kahramanmaras earthquake in 2023, Turkey. Acıbadem Üniversitesi Sağlık Bilimleri Dergisi.

[B8-behavsci-16-01897] Feo P., Di Gioia S., Carloni E., Vitiello B., Tozzi A. E., Vicari S. (2014). Prevalence of psychiatric symptoms in children and adolescents one year after the 2009 L’Aquila earthquake. BMC Psychiatry.

[B9-behavsci-16-01897] Gürsoy S., Kot M. (2026). Teaching earthquake safety skills to students with autism using video modeling. Journal of Autism and Developmental Disorders.

[B10-behavsci-16-01897] Horner R. D., Baer D. M. (1978). Multiple-probe technique: A variation on the multiple baseline. Journal of Applied Behavior Analysis.

[B11-behavsci-16-01897] Indriasari F. N., Daniyati K P., Widyarani L. (2018). Disaster risk reduction and emergency preparedness for children with autism in facing earthquake disaster in Yogyakarta. JMMR (Jurnal Medicoeticolegal dan Manajemen Rumah Sakit).

[B12-behavsci-16-01897] Kaya F., Yucesoy-Ozkan S. (2022). Self-directed video prompting with and without voice-over narration in teaching daily living skills to students with autism spectrum disorder. Education and Treatment of Children.

[B13-behavsci-16-01897] Kazdin A. E. (1982). Single case research designs: Methods for clinical and applied settings.

[B14-behavsci-16-01897] Kratochwill T. R., Hitchcock J., Horner R. H., Levin J. R., Odom S. L., Rindskopf D. M., Shadish W. R. (2010). Single-case designs technical documentation. *What Works Clearinghouse*.

[B15-behavsci-16-01897] Kubina R. M., Kostewicz D. E., King S. A., Brennan K. M., Wertalik J., Rizzo K., Markelz A. (2021). Standards of graph construction in special education research: A review of their use and relevance. Education and Treatment of Children.

[B16-behavsci-16-01897] Kurt O., Cevher Z., Kutlu M. (2024). Effectiveness of video modeling in teaching earthquake and postearthquake evacuation safety skills for children with autism. Journal of Applied Behavior Analysis.

[B17-behavsci-16-01897] Ledford J. R., Gast D. L. (2018). Single case research methodology: Applications in special education and behavioral sciences.

[B18-behavsci-16-01897] Lindsay S., Hsu S. (2024). Emergency and disaster preparedness among children and youth with disabilities and chronic conditions, their caregivers and service providers: A scoping review. Disability and Rehabilitation.

[B19-behavsci-16-01897] Liu L., Yao X., Chen J., Zhang K., Liu L., Wang G., Ling Y. (2024). Virtual reality utilized for safety skills training for autistic individuals: A review. Behavioral Sciences.

[B20-behavsci-16-01897] Lynn M. R. (1986). Determination and quantification of content validity. Nursing Research.

[B21-behavsci-16-01897] Ngoc T. B., Thu D. T., Hieu G. M., Van Cuong N., Van Viet D., Tai H. M. (2026). Adapt applied behavior analysis methods for autism spectrum disorder children in earthquake using 3D simulation. Journal of Education and Learning (EduLearn).

[B22-behavsci-16-01897] Parker R. I., Vannest K. J., Davis J. L. (2011). Effect size in single-case research: A review of nine nonoverlap techniques. Behavior Modification.

[B23-behavsci-16-01897] Pergantis P., Bamicha V., Doulou A., Christou A. I., Bardis N., Skianis C., Drigas A. (2025). Assistive and emerging technologies to detect and reduce neurophysiological stress and anxiety in children and adolescents with autism and sensory processing disorders: A systematic review. Technologies.

[B24-behavsci-16-01897] Phibbs S., Good G., Severinsen C., Woodbury E., Williamson K. (2014). What about us? Reported experiences of disabled people related to the Christchurch earthquakes. Procedia Economics and Finance.

[B25-behavsci-16-01897] Polit D. F., Beck C. T. (2006). The content validity index: Are you sure you know what’s being reported? Critique and recommendations. Research in Nursing & Health.

[B26-behavsci-16-01897] Sarı B. (2025). Disaster management following the great Kahramanmaraş earthquakes in 2023, Türkiye. Natural Hazards and Earth System Sciences.

[B27-behavsci-16-01897] Stauch T. A., Plavnick J. B. (2020). Teaching vocational and social skills to adolescents with autism using video modeling. Education and Treatment of Children.

[B28-behavsci-16-01897] Stokes T. F., Baer D. M. (1977). An implicit technology of generalization. Journal of Applied Behavior Analysis.

[B29-behavsci-16-01897] Stough L. M., Kelman I., Rodríguez H., Donner W., Trainor J. E. (2018). People with disabilities and disasters. Handbook of disaster research.

[B30-behavsci-16-01897] Şahin C. H., Kaya A. (2025). The experience of parents of children with intellectual disabilities/autism who experienced the Kahramanmaraş earthquake in Türkiye. Journal of Intellectual Disabilities.

[B31-behavsci-16-01897] Tate R. L., Perdices M., Rosenkoetter U., Shadish W., Vohra S., Barlow D. H., Horner R., Kazdin A., Kratochwill T., McDonald S., Sampson M., Shamseer L., Togher L., Albin R., Backman C., Douglas J., Evans J. J., Gast D., Manolov R., Wilson B. (2016). The single-case reporting guideline in behavioural interventions (SCRIBE) 2016 statement. Journal of School Psychology.

[B32-behavsci-16-01897] Tunç Paftalı A., Kalaycı G. Ö., Kalaycı C. (2026). “We didn’t know what to do”: Experiences of search and rescue teams with individuals with autism spectrum disorder. Humanities and Social Sciences Communications.

[B33-behavsci-16-01897] Ulaşman S., Sivrikaya T. (2025). Digital social stories for teaching earthquake safety skills to individuals with autism spectrum disorder. Journal of Autism and Developmental Disorders.

[B34-behavsci-16-01897] United Nations (2006). Convention on the rights of persons with disabilities.

[B35-behavsci-16-01897] United Nations International Strategy for Disaster Reduction (UNISDR) (2014). UNISDR 2013 survey on living with disabilities and disasters: Key findings.

[B36-behavsci-16-01897] Valenti M., Ciprietti T., Di Egidio C., Gabrielli M., Masedu F., Tomassini A. R., Sorge G. (2012). Adaptive response of children and adolescents with autism to the 2009 earthquake in L’Aquila, Italy. Journal of Autism and Developmental Disorders.

[B37-behavsci-16-01897] Vannest K. J., Ninci J. (2015). Evaluating intervention effects in single-case research designs. Journal of Counseling & Development.

[B38-behavsci-16-01897] Vannest K. J., Parker R. I., Gonen O., Adiguzel T. (2011). Single case research: Web-based calculators for SCR analysis *(version 1.0) [Computer software]*.

[B39-behavsci-16-01897] Villeneuve M., Abson L., Pertiwi P., Moss M. (2021). Applying a person-centred capability framework to inform targeted action on disability inclusive disaster risk reduction. International Journal of Disaster Risk Reduction.

[B40-behavsci-16-01897] What Works Clearinghouse (2020). What Works Clearinghouse standards handbook, Version 4.1.

[B41-behavsci-16-01897] Wolf M. M. (1978). Social validity: The case for subjective measurement or how applied behavior analysis is finding its heart. Journal of Applied Behavior Analysis.

[B42-behavsci-16-01897] World Medical Association (2013). World Medical Association Declaration of Helsinki: Ethical principles for medical research involving human subjects. JAMA.

